# Impact of immediate and delayed chilling of raw milk on chemical changes in lipid fraction of pasteurized milk

**DOI:** 10.1186/s12944-018-0843-0

**Published:** 2018-08-17

**Authors:** Muhammad Ajmal, Muhammad Nadeem, Muhammad Imran, Muhammad Abid, Maryam Batool, Imran Taj Khan, Nabila Gulzar, Muhammad Tayyab

**Affiliations:** 1 Department of Dairy Technology, University of Veterinary and Animal Sciences, Lahore, Pakistan; 2 Institute of Home and Food Sciences, Faculty of Life Sciences, Government College University, Faisalabad, Pakistan; 3 Department of Dairy Technology, PMAS, Arid Agriculture University Sub-Campus, Khushab, Pakistan; 4 Institute of Biochemistry and Biotechnology, University of Veterinary and Animal Sciences, Lahore, Pakistan

**Keywords:** Raw milk, Delay chilling, Lipids characterization, Pasteurization, Storage

## Abstract

**Background:**

In many developing countries, milk chilling facilities are not available on the farm where milk is produced, rather these are located at the distance of 10–12 km. After milking, it takes about 2–3 h to reach milk to the chilling facilities. The milk is then chilled and transported to the milk processing plants for thermal processing and value addition. In developing countries, shelf life of pasteurized milk is only 3 days, as compared to 7–10 days in developed countries. The factors which are responsible for the shorter shelf life of pasteurized milk should be discovered for the improvement of dairy sectors of these countries. The magnitude of chemical changes which takes place in un-chilled milk and their effect on fatty acids profile, antioxidant status and lipid oxidation is not previously studied.

**Methods:**

Raw milk samples of the same farm were either rapidly chilled to 4 °C immediately or held at room temperature (35 ± 2 °C) for 2 h followed by rapid chilling to 4 °C. Immediately and delayed chilled raw milk samples were stored at 4 °C for 72 h. Both milk samples were pasteurized at 65 °C, filled in 250 ml transparent PET bottles and stored at 4 °C for 6 days. Fatty acid profile, selenium, zinc, total antioxidant capacity, total flavonoid content and 1, 1-diphenyl-2-picrylhydrazyl (DPPH) free radical scavenging activity, free fatty acids, peroxide value and anisidine value were determined at different stages of the experiment. This experiment was repeated with milk of same farm for at least five times.

**Results:**

Storing raw milk at ambient temperature (35 ± 2 °C) significantly influenced the pH and lactose content of milk. The loss of short-chain fatty acids in delayed chilled milk was 1.19%, 3.27% and 1.60%, as compared to immediately chilled raw milk. In delayed chilled milk, loss of C_18:1_ and C_18:2_ after 3 days of storage period was 6.67% and 01.22. In delayed chilled milk after 6 days of storage, loss of C_18:1_ and C_18:2_ was 7.7% and 1.39%, respectively. In immediately chilled milk loss of C_18:1_ and C_18:2_ after 3 days of storage was 3.48% and 0.64%. In immediately chilled milk loss of C_18:1_ and C_18:2_ after 6 days of storage was 4.57% and 0.9%. Almost 41% vitamin E was lost when raw milk was stored at ambient temperature for 2 hrs. About 21% and 7% vitamin E was lost in delayed and immediately chilled milk, when samples were analyzed immediately after pasteurization. Loss of selenium and zinc contents after 2 h of ambient storage of raw milk were 0.43 and 224 μg/100 g. After 2 h of storage of milk at ambient temperature, free fatty acids increased by 0.03% (*p* < 0.05). After 6 days of storage, rise of free fatty acids in immediately and delayed chilled milk was 0.06% and 0.14%, respecitively. Rise of 0.13(MeqO_2_/kg) was recorded, when un-chilled raw milk was stored at ambient temperature for 2 h. After 3 and 6 days of storage, peroxide value of pasteurized milk (delayed chilled) was 0.88 and 1.56 (MeqO_2_/kg). After 3 and 6 days of storage, peroxide value of pasteurized (immediately chilled) was 0.39 and 0.42(MeqO_2_/kg). After 2 hrs of ambient storage, 18.41% flavonoids were lost. After 2 hrs of ambient storage of raw milk, loss of total antioxidant capacity and DPPH free radical scavenging activity was 29.31% and 44.53%. After 6 days of pasteurization, loss of total antioxidant capacity and DPPH free radical scavenging activity in delayed chilled raw milk was 72.1% and 89.57%.

**Conclusions:**

The findings of this investigation showed that delayed chilling of raw milk leads to several undesirable chemical changes in lipid fraction of milk.

## Background

In many Asian and African countries, most of the milk is produced on small farms. On an average basis, about 10–15 l milk is produced on each farm. Chilling facilities are usually installed to cater the milk of about 15–25 villages. The distance between the place of milk production and milk chilling facilities is about 10–12 KM. On an average basis, it takes about 2 h to reach milk from the point of production to the chilling facility. During this period, several chemical changes take place in raw milk. Due to perishable nature of milk, immediate chilling of raw milk on the farm is recommended, however, due to several reasons in developing countries, practically it is impossible to chill the milk on the farm. To increase the shelf life of raw milk, below 4 °C chilling is recommended and commonly adopted practice all over the world. In Europe, milk processing companies collect chilled milk after every 3 days. Chilled milk may undergo several biochemical changes, certain species of the genus bacillus and *pseudomonas fluorescens* can produce proteases and lipases, these enzymes have the capability of hydrolyzing fat and proteins of milk. During the storage of raw milk at lower temperature, change in microflora of milk takes place, when population of psychrophilic bacteria reaches about 10^7^ to 10^8^/ml [1]. During the storage of heat treated milk, proteases and lipases induces several undesirable flavors such as bitter, foreign, unclean, fruity, yeasty, metallic and bitter flavor [2]. Development of oxidized flavors in pasteurized milk is generally a result of bacterial growth [3]. Unpleasant aroma in pasteurized milk is caused by lipolysis of fluid milk after pasteurization by growth of psychrophilic bacteria in raw milk [4]. Indigenous and bacterial lipases may lead to the hydrolysis of fat globule, the degree of free fatty acid production in pasteurized milk depends upon psychrophilic count, storage temperature and metal ions etc. [5]. Objectionable flavors in raw milk may also be due to the generation of free fatty acids [6]. Low temperature/ chilling induced biochemical changes in raw milk during the 3 days of storage period should be studied for better quality and shelf life of milk and dairy products, as this aspect is not previously investigated. Thermal treatment is mandatory for the manufacturing of fluid milk and other dairy products, the most commonly used thermal technique is pasteurization [7]. Pasteurized milk requires refrigeration during distribution/ storage and offers wide range of products for the dairy industry [8]. Pasteurization and subsequent storage of milk may induce some undesirable biochemical changes in lipid fraction of milk e.g. hydrolysis and auto-oxidation, these undesirable changes lead to the lower consumer acceptability and shorter shelf life [9]. Heat resistant bacterial lipases are one of the most common cause of spoilage of spoilage milk [10]. Milk fat is considered as one of the most significant milk constituent with respect to the variety of fatty acids, about 400 different fatty acids have been identified in milk [11]. Thermal processing may influence the physical and chemical characteristics of milk fat such production of *trans* isomers and auto-oxidation. Auto-oxidation in milk fat consequences in the generation of low molecular weight aldehydes, ketone and lactones [12]. These low molecular weight substances induce offensive odor and reduce the amount of fat soluble vitamins in milk [13]. Few oxidation products are connected with the damage of cellular membrane, ageing, cancer and heart diseases [14, 15]. Biochemical changes taking place in fat fraction of pasteurized milk needs more detailed investigation. In few studies, the effect of heat treatment on lipolysis of milk is described. In these studies, decline in total fat content of the milk was used as indication of lipolysis [16]. Effect of heat treatment on some chemical perspectives of pasteurized milk has been studied in detail. However, the effect of delayed/ immediate chilling, 72 hours of chilling on biochemical changes in lipid fraction of pasteurized milk needs more detailed investigation. This study was planned with the objective to determine the effect of immediate/ delayed chilling, 72 h of chilling on fatty acid profile and lipid oxidation in pasteurized milk using conventional and advanced analytical techniques.

## Methods

### Materials and experimental plan

Raw milk was obtained from a farm and all the milk producing animals were in good health. For hygienic milk collection, sampler and transparent glass bottles were sterilized. Raw milk obtained from a farm was divided into two parts and packaged in sterilized transparent glass bottles. One part was immediately cooled down to 4 °C using chilled water (2 °C) designated as immediately chilled milk, second part was allowed to stand at room temperature (35 °C) for 2 hrs, then it was also cooled down to 4 °C using chilled water (2 °C), designated as delayed chilled milk. Both type of milk was subjected to 72 h chilling at 4 °C, followed by pasteurization at 65 °C for 30 min, filled in 250 ml transparent sterilized bottles and stored at 4 °C for 6 days.

### Chemical composition of milk

Chemical composition of milk samples was determined on a lactoscan. Fat, protein, lactose content and pH were determined.

### Estimation of fatty acids profile

For the estimation of fatty acid profile of milk, first fat was extracted using diethyl ether, 50–60 mg sample was weighed in screw capped test tube with the help of digital micropipette, followed by addition of 3 ml 2, 2, 4 trimethyl pentane then 0.5 N sodium methoxide solution prepared in HPLC grade methanol was added, samples were vortex at 1500 Rpm for 3 min, followed by 15 min staying time. Upper layer was transferred to GC vials and 1 μl was injected into GC-MS (Agilent, 7890-B) through auto-sampler, fitted with a methyl lignoserate-coated (film thickness 0.25 m), SP-2330 (SUP ELCO Inc. Supelco Park Bellefonte, PA 16823–004 8, USA) polar capillary column (30 m × 0.32 mm). Peaks were identified and quantified by internal standards (FAME-37) [17, 18].

### Total flavonoid content

Estimation of total flavonoid content in milk samples was performed by a spectrophotometric method using AlCl_3_ as derivatizing agent. For this test, Rutin was used as standard, milk sample 0.1 ml and 5% solution of NaNO3 (0.2 ml) were mixed together and incubated for 5 min, then 1 M NaOH 1 mL and 0.2 mL AlCl_3_ were added and samples were incubated for 15 min at room temperature. For the measurement of absorbance, spectrophotometer was used (510 nm) [19].

### Total antioxidant capacity

Total antioxidant capacity was determined in terms of Ascorbic Acid Equivalent/g [20]. Absorbance of the sample, blank and series of standards was recorded at 695 nm.

### 1, 1-diphenyl-2-picrylhydrazyl (DPPH) free radical scavenging activity

Sample (1 ml) was mixed with DPPH solution, which was prepared in in methanol, contents of the test tube were mixed by vortexing followed by incubation at 25 °C for 20 min. Double beam spectrometer was used for determined absorbance at 517 nm and results were reported in percent inhibition of free radicals [21].

### Estimation of vitamins

For the extraction of fat from milk, standard method was adopted [22]. For the estimation tocopherols, 200 μg sample was homogenized with *n*-hexane (1 ml), sample was injected into HPLC (Supelco, Bellefonte, PA). For the preparation of mobile phase, acetic acid and ethyl acetate (0.5% both) in n-hexane, flow rate was 1.5 ml/min, results of tocopherol were reported in μg/g [23]. For the estimation of vitamin A, milk sample (25 ml) was blended 20 ml each ammoni and ethanol ammonia (25% and 96%). BHT was added to the upper layer at the rate of 0.0025% as an antioxidant, followed by drying the sample at 35 °C using rotary evaporator, 30 ml KOH (5% in ethanol) was added, saponified at 60 °C for 30 min, extracted with *n*-hexane, evaporated on a rotary evaporator. Measurement was performed on HPLC using retinol acetate as standard, mobile phase was composed of acetonitrile-methanol 85:15 [24]. For the determination of vitamin C, each 300 μl milk and metaphosphoric (0.56%) were mixed together. Absorbance was measured at 254 nm against ascorbic acid standard [25].

### Lipid oxidation

For the measurement of lipid oxidation in milk at different stages of storage and processing, free fatty acids, peroxide value and anisidine value was determined for 6 days at the frequency of 0, 3 and 6 days using standard methods [26].

### Determination of zinc and selenium

Selenium and Zinc were determined by the standard methods [27].

### Statistical analysis

Experiment was performed in a completely randomized design; each treatment was replicated three times and every sample was analyzed for three times. Data was analyzed using two-way analysis of variance technique. For the determination of significant difference, Duncan Multiple Range Test was used using SAS 9.1 software [28].

## Results and discussion

### Chemical composition of milk

Table 1 describes the chemical composition of raw, chilled, pasteurized, 3 and 6 days old milk. Delayed chilling, 72 h of chilling and pasteurization treatment did not have any impact on fat content of milk, however, storage had a significant effect on fat content. After 6 days of storage, fat and protein content of delayed chilled raw milk were significantly less than their initial values (*p* < 0.05). Delayed chilling of raw milk also had a significant impact on lactose content and pH of milk. Hassan et al. [29] studied the physical and chemical characteristics of heat treated milk during the storage, fat, protein and SNF content while significant changes were reported in pH of heat treated milk after 12 during storage. Pasteurization did not have a significant impact on lactose and protein content of milk, while, protein content and acidity were significantly affected by the storage [30]. AlKanhal et al. [31] studied the effect of storage on compositional attributes of milk, they recorded a decline in protein content with the progression of storage period.

**Table 1:** Effect of Immediate and Delayed Chilling of Raw Milk on Chemical Composition of Pasteurized Milk

Stage of Sampling	Fat%	Protein%	Lactose%	pH	TS%
Raw Milk (Immediately Chilled After Milking)	4.32 ± 0.04^a^	3.22 ± 0.08^a^	4.62 ± 0.08^a^	6.71 ± 0.20^a^	13.2 ± 0.03^a^
Raw Milk Chilled After 2 Hrs of Storage (32 ± 1 °C)	4.29 ± 0.03^a^	3.18 ± 0.06^a^	4.55 ± 0.06^b^	6.61 ± 0.05^b^	13.1 ± 0.11^a^
Raw Milk Chilled for 72 Hrs (Immediately Chilled Milk)	4.30 ± 0.02^a^	3.21 ± 04^a^	4.61 ± 0.5^a^	6.69 ± 0.03^a^	13.2 ± 0.08^a^
Raw Milk Chilled for 72 Hrs (Delayed Chilled Milk)	4.28 ± 0.05^a^	3.18 ± 0.06^a^	4.55 ± 0.03^b^	6.60 ± 0.02^b^	13.1 ± 0.09^a^
0 Day After Pasteurization (Immediately Chilled Milk)	4.27 ± 0.06^a^	3.21 ± 0.07^a^	4.60 ± 0.06^a^	6.61 ± 0.05^b^	13.2 ± 0.07^a^
0 Day After Pasteurization Treatment (Delayed Chilled Milk)	4.28 ± 0.04^a^	3.18 ± 0.05^a^	4.54 ± 0.05^b^	6.67 ± 0.04^a^	13.1 ± 0.05^a^
After 3 Days of Storage (Immediately Chilled Milk)	4.25 ± 0.08^a^	3.19 ± 0.03^a^	4.59 ± 0.04^a^	6.66 ± 0.03^a^	13.2 ± 0.08^a^
After 3 Days of Storage (Delayed Chilled Milk)	4.24 ± 0.7^a^	3.17 ± 0.08^a^	4.51 ± 0.02^b^	6.58 ± 0.07^b^	13.1 ± 0.06^a^
After 6 Days of Storage (Immediately Chilled Milk)	4.26 ± 0.05^a^	3.16 ± 0.06^a^	4.57 ± 0.03^a^	6.62 ± 0.05^a^	13.0 ± 0.03^a^
After 6 Days of Storage (Delayed Chilled Milk)	4.20 ± 0.09^b^	2.95 ± 0.09^b^	4.52 ± 0.06^b^	6.54 ± 0.06^b^	12.7 ± 0.02^b^

In a column, if a mean is expressed by dissimilar letter, these are statistically significant (*p* < 0.05)

### Fatty acid profile

Milk fat is a highly complex fat, more 200 types of fatty acids have been recognized in it, it is the most common and abundant source of short-chain fatty acids. Specific flavour and taste perspectives of dairy products are mainly due to the short-chain fatty acids. In addition to that, milk fat is also a reasonable source of oleic acid, which is regarded beneficial fatty acid in prevention of dyslipidaemia. Milk fat also contains some concentration of linoleic acid (C_18:2_). Unsaturated fatty acids are usually susceptible to auto-oxidation [32]. Auto-oxidation of unsaturated fatty acids also depend upon the storage temperature, it is recommended that raw milk should be immediately stored after milking, quality of processed milk and dairy products largely depends upon the quality of raw milk. In current investigation, storage of un-chilled raw milk for 2 h considerably influenced the fatty acid profile, major transition was recorded in short, medium and long-chain fatty acids. Concentrations of short, medium and long-chain unsaturated fatty acids in immediately chilled raw milk were 10.4%, 44.97% and 26.42%. While the concentration of short, medium and long-chain unsaturated fatty acids in delayed chilled (raw milk chilled after 2 h of ambient storage) were 9.21%, 41.7% and 24.82%. The decline in the concentration of short-chain fatty acids in delayed chilled milk was 1.19%, 3.27% and 1.60%, as compared to immediately chilled raw milk (Table 2). Functional properties of milk fat are mainly due to the medium-chain fatty acids [33]. Decline in the concentration of medium-chain fatty acids may also lead to the downing of desirable attributes in value added milk products prepared from milk with lower magnitude of medium-chain fatty acids. Vazquez-Landaverde et al. [34] found that C_16:0_, C_18:0_ and C_18:1_ were the major fatty acids in milk. Oleic acid was the major unsaturated fatty acids in milk [35]. Nadeem et al. [36] prepared ice cream from the milk which have a lower concentration of medium-chain fatty acids, ice cream had a week texture as compared to the ice cream prepared from milk fat with normal fatty acid composition. Chilling and pasteurization of milk did not have any significant impact on fatty acid profile of immediately and delayed chilled milk. In both types of pasteurized milks, concentration of short-chain and medium chain fatty acid increased while concentration of unsaturated fatty acids decreased. Effect of heat treatment on fatty acid profile of milk is reported in literature Khan et al. [37] studied the impact of pasteurization and boiling on fatty acid profile of cow and buffalo milk, they reported that heat treated cow and buffalo milk had higher concentration of short and medium-chain fatty acids with lower amount of long-chain unsaturated fatty acids. Concentration of short and medium chain fatty acids in heat treated milk is lower than raw milk [38]. Storage period significantly affected the fatty acid profile of delayed chilled milk, while non-significant changes were recorded in the fatty acid profile of immediately chilled milk. In delayed chilled milk, loss of C_18:1_ and C_18:2_ after 3 days of storage period was 6.67% and 01.22. In delayed chilled milk after 6 days of storage, loss of C_18:1_ and C_18:2_ was 7.7% and 1.39%, respectively. In immediately chilled milk loss of C_18:1_ and C_18:2_ after 3 days of storage was 3.48% and 0.64%. In immediately chilled milk loss of C_18:1_ and C_18:2_ after 6 days of storage was 4.57% and 0.9%. Amount of unsaturated fatty acids decreased during the storage period [39]. Ullah et al. [40] found that concentration of unsaturated fatty acids in milk fat decreased during the long-term storage. Nadeem et al. [32] also observed the same trend in fatty acid profile, when milk fat was stored for 6 months. It was noted that storing of raw milk at ambient temperature for 2 h altered the fatty acid profile. Fatty acids were broken down to oxidation products therefore, peroxide value and anisidine value of delayed chilled at all the subsequent stages were higher than immediately chilled milk. From the fatty acid profile of milk at different stages of storage and processing, it is evident that delayed chilling of raw milk is the major reason for undesirable changes in lipid fraction of pasteurized milk.

**Table 2:** Effect of Immediate and Delayed Chilling of Raw Milk on Fatty Acid Profile of Pasteurized Milk

Fatty Acid	Raw Milk (Immediately Chilled After Milking)	Raw Milk Chilled After 2 Hrs of Storage (32 ± 1 °C)	Raw Milk Chilled for 72 Hrs (Immediately Chilled Milk)	Raw Milk Chilled for 72 Hrs (Delayed Chilled Milk)	0 Day After Pasteurization (Immediately \Chilled Milk)	0 Day After Pasteurization Treatment (Delayed Chilled Milk)	After 3 Days of Storage (Immediately Chilled Milk)	After 3 Days of Storage (Delayed Chilled Milk)	After 6 Days of Storage (Immediately Chilled Milk)	After 6 Days of Storage (Delayed Chilled Milk)
C_4:0_	3.48 ± 0.92^d^	3.11 ± 0.09^e^	3.46 ± 0.5^d^	3.39 ± 0.08^d^	3.15 ± 0.07^e^	3.72 ± 0.08^c^	4.12 ± 0.9^a^	3.51 ± 0.25^c^	3.95 ± 0.3^b^	3.11 ± 0.09^e^
C_6:0_	2.38 ± 0.37^d^	2.19 ± 0.07^e^	2.35 ± 0.3^c^	2.28 ± 0.11^d^	2.27 ± 0.11^d^	2.52 ± 0.12^b^	3.08 ± 0.16^a^	2.39 ± 0.11^c^	2.31 ± 0.02^d^	2.16 ± 0.06^e^
C_8:0_	1.41 ± 0.14^c^	1.24 ± 0.01^e^	1.39 ± 0.06^c^	1.31 ± 0.05^d^	1.31 ± 0.03^d^	1.66 ± 0.07^b^	1.78 ± 0.02^a^	1.42 ± 0.06^c^	1.61 ± 0.03^b^	1.22 ± 0.06^e^
C_10:0_	3.13 ± 0.24^d^	2.67 ± 0.03^f^	3.11 ± 0.04^d^	2.95 ± 0.12^e^	2.91 ± 0.08^e^	3.55 ± 0.09^b^	3.62 ± 0.08^a^	3.36 ± 0.07^c^	3.53 ± 0.05^b^	2.96 ± 0.01^e^
C_12:0_	3.56 ± 0.16^d^	3.17 ± 0.04^f^	3.55 ± 0.05^d^	3.21 ± 0.04^f^	3.61 ± 0.12^d^	3.84 ± 0.10^b^	4.10 ± 0.15^a^	3.42 ± 0.03^e^	3.77 ± 0.12^c^	3.24 ± 0.03^f^
C_14:0_	11.63 ± 0.46^b^	10.16 ± 0.01^c^	11.59 ± 0.3^b^	10.67 ± 0.06^c^	11.25 ± 0.14^b^	11.51 ± 0.03^b^	12.17 ± 0.43^a^	10.64 ± 0.31^c^	11.38 ± 0.5^b^	9.73 ± 0.09^d^
C_16:0_	29.78 ± 1.35^b^	28.37 ± 0.14^c^	29.72 ± 0.7^b^	28.75 ± 0.37^c^	29.11 ± 0.07^b^	31.35 ± 0.08^a^	31.98 ± 0.16^a^	30.15 ± 0.58^b^	29.10 ± 0.38^b^	28.43 ± 0.88^c^
C_18:0_	13.77 ± 0.80^a^	12.55 ± 0.12^b^	13.66 ± 0.4^a^	12.85 ± 0.14^b^	12.78 ± 0.05^b^	10.27 ± 0.64^d^	11.22 ± 0.31^c^	9.60 ± 0.24^e^	8.85 ± 0.22^f^	8.62 ± 0.36^f^
C_18:1_	24.89 ± 1.30^a^	23.61 ± 0.06^b^	24.85 ± 0.6^a^	23.57 ± 0.25^b^	21.47 ± 0.13^c^	19.76 ± 0.49^e^	21.41 ± 0.72^c^	18.22 ± 0.45^e^	20.32 ± 0.61^d^	17.19 ± 0.20^f^
C_18:2_	1.53 ± 0.19^a^	1.21 ± 0.02^c^	1.49 ± 0.04^a^	1.29 ± 0.03^b^	0.88 ± 0.04^c^	0.43 ± 0.01^d^	0.84 ± 0.02^c^	0.31 ± 0.03^f^	0.63 ± 0.02^e^	0.14 ± 0.01^g^
C_18:3_	0.49 ± 0.01^a^	0.27 ± 0.01^b^	0.48 ± 0.01^a^	0.26 ± 0.01^b^	0.19 ± 0.01^c^	0.12 ± 0.02^d^	0.16 ± 0.01^c^	0.08 ± 0.01^f^	0.11 ± 0.01^e^	ND

In a row, if a mean is expressed by dissimilar letter, these are statistically significant (*p* < 0.05)

*ND* Not Detected

### Transition in vitamins

Milk has two different types of antioxidant systems, these are categorized as fat soluble and water-soluble antioxidant system. Vitamin A, E and carotenoids constitute the fat-soluble antioxidant system of milk while, vitamin C, tyrosine, casein, whey proteins, zinc and selenium etc. constitute the water-soluble antioxidant system. These antioxidant defense system can prevent oxidative stresses in the body, they can also help to inhibit the lipid oxidation in milk [33]. Estimation of variation in vitamin content of the immediately chilled raw milk, delayed chilled milk, raw milk chilled for 72 h, freshly pasteurized milk, 3 and 6 days old pasteurized milk may provide a useful evidence about the antioxidant behaviour of milk at these stages, therefore, these parameters were studied in this study using HPLC. Vitamin A content of immediately chilled raw milk was 0.46 μg/100 g, about 41% vitamin E was lost when raw milk was stored at ambient temperature for 2 hrs. Vitamin A content of immediately and delayed chilled milk were not affected by the chilling of raw milk for 72 h. About 21% and 7% vitamin E was lost in delayed and immediately chilled milk, when samples were analyzed immediately after pasteurization. After 3 days of storage of pasteurized milk, loss of vitamin E in immediately and delayed chilled milk was 17.39%, after 3 days of storage of pasteurized milk, loss of vitamin E in delayed and immediately chilled raw milk was 76.1% as compared to the vitamin A content of raw milk (immediately chilled after milking). After 6 days of storage of pasteurized milk, loss of vitamin E in delayed and immediately chilled raw milk was 91.3% and 36.9% (Table 3). Ohlsson and Bengtsson [41] monitored the changes in vitamin A content, when milk was subjected to heat treatment, it was found that heat treatment had a little effect on vitamin A content of the milk. Saffert et al. [42] recorded a slight decrease in the amount of vitamin A of heat treated milk. Vitamin A content of the raw milk subjected to immediate chilling were 0.63 mg/100 g, while storing of milk at ambient temperature for 2 hrs resulted in decline of 41.2% vitamin E. Losses of folic acid may be 50% in sterilized milk [43]. Chilling of both types of raw milks (immediately and delayed chilled) did not have any significant effect on vitamin E content. Pasteurization had a varying degree of impact on vitamin E content of milk, loss of vitamin E immediately chilled milk was only 5% while, 12% vitamin E was lost in delayed chilled milk. Effect of storage period on vitamin E content of pasteurized milk was also different for immediately and delayed chilled raw milk. After 3 days of storage of pasteurized milk (immediately chilled milk), loss of vitamin E in pasteurized milk was 21%, from the initial value of immediately chilled raw milk. After 3 days of storage of pasteurized milk (delayed chilled milk), loss of vitamin E pasteurized was 52%, from the initial value of immediately chilled raw milk. After 6 days of storage of pasteurized milk (immediately chilled milk), loss of vitamin E in pasteurized milk was 30%, from the initial value of immediately chilled raw milk. After 6 days of storage of pasteurized milk (delayed chilled milk), loss of vitamin E in pasteurized milk was 90.4%, from the initial value of immediately chilled raw milk. Vitamin C content of raw milk was 0.57 mg/100 g, amount of vitamin C significantly decreased in 2 hrs stored raw milk at ambient temperature. Storing the raw milk at ambient temperature leads to several chemical and biochemical changes which leads to the reduction of vitamin in the subsequent stages of processing and storage, it was not detected in 6 days old pasteurized milk samples (delayed chilled raw milk).

**Table 3:** Effect of Immediate and Delayed Chilling of Raw Milk on Vitamins and Mineral Content of Pasteurized Milk

Stage of Sampling	Vitamin A μg/100 g	α-Tocopherol mg/100	Vitamin C mg/100 g	Selenium μg/100 g	Zinc μg/100 g
Raw Milk (Immediately Chilled After Milking)	0.46 ± 0.02^a^	0.63 ± 0.06^a^	0.57 ± 0.05^a^	3.29 ± 0.04^a^	4735 ± 0.60^a^
Raw Milk Chilled After 2 Hrs of Storage (32 ± 1 °C)	0.27 ± 0.01^b^	0.37 ± 0.02^d^	0.26 ± 0.03^d^	2.86 ± 0.05^c^	4511 ± 0.44^c^
Raw Milk Chilled for 72 Hrs (Immediately Chilled Milk)	0.44 ± 0.05^a^	0.61 ± 0.03^a^	0.54 ± 0.06^a^	3.28 ± 0.03^a^	4726 ± 0.66^a^
Raw Milk Chilled for 72 Hrs (Delayed Chilled Milk)	0.24 ± 0.04^d^	0.34 ± 0.02^d^	0.23 ± 0.01^d^	2.85 ± 0.02^c^	4506 ± 0.53^c^
0 Day After Pasteurization (Immediately Chilled Milk)	0.41 ± 0.08^a^	0.51 ± 0.06^b^	0.38 ± 0.02^b^	3.25 ± 0.05^a^	4722 ± 0.64^a^
0 Day After Pasteurization Treatment (Delayed Chilled Milk)	0.19 ± 0.03^e^	0.25 ± 0.03^e^	0.13 ± 0.04^d^	2.81 ± 0.02^c^	4491 ± 0.55^d^
After 3 Days of Storage (Immediately Chilled Milk)	0.38 ± 0.07^b^	0.42 ± 0.04^c^	0.31 ± 0.01^c^	3.23 ± 0.03^a^	4714 ± 0.67^a^
After 3 Days of Storage (Delayed Chilled Milk)	0.11 ± 0.02^f^	0.11 ± 0.01^f^	0.05 ± 0.02^e^	3.06 ± 0.01^b^	4364 ± 0.52^e^
After 6 Days of Storage (Immediately Chilled Milk)	0.29 ± 0.03^c^	0.33 ± 0.04^e^	0.20 ± 0.01^d^	3.19 ± 0.03^a^	4695 ± 0.63^b^
After 6 Days of Storage (Delayed Chilled Milk)	0.04 ± 0.01^g^	0.06 ± 0.02^g^	Not Detected	2.74 ± 0.05^c^	4239 ± 0.49^f^

In a column, if a mean is expressed by dissimilar letter, these are statistically significant (*p* < 0.05)

### Selenium and zinc

Selenium and zinc are micro mineral which are naturally present in milk, these have antioxidant activity in milk. Research work has shown that pasteurization usually have no effect on total mineral content of milk, however, information regarding the changes in content of selenium and zinc is scarce. This is the first investigation in which effect processing and storage is determined on selenium and zinc content of milk. Siddique et al. [44] found non-significant effect of heat treatment and storage on total mineral content of milk. Selenium and zinc contents of immediately raw chilled raw milk were 3.29 and 4735 μg/100 g. Loss of selenium and zinc after 2 h of ambient storage of raw milk were 0.43 and 224 μg/100 g (Table 3). Chilling and pasteurization have non-significant effect on selenium and zinc content of immediately and delayed chilled milk. Effect of storage on selenium and zinc on both immediately and delayed chilled milk up to 3 days was non-significant. After 6 days of storage, loss of selenium and zinc in immediately and delayed chilled milk were 0.55 and 496 μg/100 g. Selenium is an essential mineral, as per dietary guidelines, 50 μg/day selenium should be consumed on daily basis to prevent cardiovascular diseases and cancer etc. [45]. Zinc is a major antioxidant mineral of milk, it is an essential mineral, it should be consumed at the rate of 15 mg/day. Literature reported the use of selenium for the supplementation of dairy products [46].

### Lipid oxidation

For the estimation of lipid oxidation in raw and pasteurized milk, free fatty acids, peroxide value and anisidine value were analyzed. Free fatty acids are the indicators of hydrolytic rancidity, higher concentration of free fatty acids in milk shows the excessive lipase activity in milk. While, peroxide value and anisidine value quantifies the peroxides and aldehydes especially 2-alkenals [47]. As per standards of European Union, maximum allowable limit of free fatty acids, peroxide value and anisidine value is 0.2%, 10MeqO_2_/kg and 10, respectively. It is practically observed that foods having free fatty acids more than 0.16% usually have lower shelf stability and peroxide value of more than 1MeqO_2_/kg usually reveals give a slight rancid flavour in foods [33]. Free fatty affects the fats and oils in two ways, firstly, they lead to the generation of odoriferous compounds. Secondly, they lead to the acceleration of auto-oxidation, resulting in shorter shelf of fats and oils [48]. After 2 h of storage of milk at ambient temperature, free fatty acids increased by 0.03% (*p* < 0.05). Free fatty acids of 72 h chilled milk (immediately chilled raw milk) were not different from the free fatty acids of milk immediately chilled milk (Table 4). Pasteurization did not have any pronounced effect on free fatty acids content of both delayed and immediately chilled milk. After 3 days of storage, rise of free fatty acids in immediately chilled and delayed chilled milk was 0.01% and 0.04%. After 6 days of storage, rise of free fatty acids in immediately and delayed chilled milk was 0.06% and 0.14%. Keeping in view the influence of free fatty acids on sensory and storage perspectives, edible oil manufacturer strives to a free fatty acid in the range of 0.08% to 0.11%. Free fatty acids of immediately chilled raw milk after 6 days of pasteurization were 0.11%. Role of free fatty acids in the acceleration of auto-oxidation, induction of bad flavours etc. is well established, however, the effect of delayed chilling of raw milk on free fatty acids was determined in this investigation. The rise of 0.14% free fatty acids during 6 days of storage of delayed chilled raw milk was due to the production of more lipases during the 2 hrs of ambient storage, lipase activity in milk stored at ambient temperature without chilling was also significantly higher than the immediately chilled raw milk. In milk lipases may be originated from milk and bacteria, in 2 hrs time, total plate count significantly increased that leads to excessive lipase activity. Lipases of bacterial origin are more resistant to heat treatment, they survive the pasteurization treatment and may cause / speed up the spoilage of pasteurized milk [4]. Peroxide value is regarded is one the most reliable and simple method for the assessment of oxidation status of fats and oils, however, this method is not frequently to know the oxidation status of raw and pasteurized milk. Rise of 0.13(MeqO_2_/kg) was recorded, when un-chilled raw milk was stored at ambient temperature for 2 h. Chilling (72 h) and pasteurization did not impart any noticeable impact on peroxide value. However, storage period had a major effect on peroxide value of delayed chilled at both testing intervals. After 3 and 6 days of storage, peroxide value of pasteurized milk (delayed chilled) was 0.88 and 1.56 (MeqO_2_/kg). After 3 and 6 days of storage, peroxide value of pasteurized (immediately chilled) was 0.39 and 0.42(MeqO_2_/kg). Peroxide value also predict the shelf life of foods and fat rich dairy products [32]. Milk sample having peroxide value of 0.35 at the time of chilling and immediately after pasteurization 0.48(MeqO_2_/kg) revealed poor storage stability as compared to the milk sample having 0.22 peroxide value at the time of chilling and 0.24 (MeqO_2_/kg) immediately after pasteurization. In developing countries, raw milk takes few to many hrs to reach the chilling facility, therefore, the shelf life of pasteurized milk is low as compared to the developed countries, where milk is immediately cooled after milking. Anisidine value of delayed chilled raw milk was also higher than immediately chilled milk at all stages of processing and storage (Table 4).

**Table 4:** Effect of Immediate and Delayed Chilling of Raw Milk on Lipid Oxidation of Pasteurized Milk

Stage of Sampling	FFA% (Oleic Acid)	Peroxide Value (MeqO_2_/kg)	Anisidine Value
Raw Milk (Immediately Chilled After Milking)	0.05 ± 0.01^e^	0.22 ± 0.03^f^	2.58 ± 0.8^e^
Raw Milk Chilled After 2 Hrs of Storage (32 ± 1 °C)	0.08 ± 0.02^d^	0.35 ± 0.05^d^	3.64 ± 0.09^c^
Raw Milk Chilled for 72 Hrs (Immediately Chilled Milk)	0.05 ± 0.01^e^	0.24 ± 0.03^e^	2.59 ± 0.07^e^
Raw Milk Chilled for 72 Hrs (Delayed Chilled Milk)	0.11 ± 0.06^c^	0.36 ± 0.06^d^	3.66 ± 0.10^c^
0 Day After Pasteurization (Immediately Chilled Milk)	0.08 ± 0.03^d^	0.24 ± 0.04^e^	2.65 ± 0.04^e^
0 Day After Pasteurization Treatment (Delayed Chilled Milk)	0.12 ± 0.07^c^	0.48 ± 0.08^c^	3.72 ± 0.07^c^
After 3 Days of Storage (Immediately Chilled Milk)	0.09 ± 0.02^d^	0.39 ± 0.06^d^	2.74 ± 0.08^e^
After 3 Days of Storage (Delayed Chilled Milk)	0.16 ± 0.04^b^	0.88 ± 0.09^b^	4.14 ± 0.11^b^
After 6 Days of Storage (Immediately Chilled Milk)	0.11 ± 0.06^c^	0.42 ± 0.05^c^	3.11 ± 0.12^d^
After 6 Days of Storage (Delayed Chilled Milk)	0.19 ± 0.07^a^	1.56 ± 0.11^a^	7.89 ± 0.50^a^

In a column, if a mean is expressed by dissimilar letter, these are statistically significant (*p* < 0.05)

### Storage effect on antioxidant capacity of pasteurized milk

Effect of pasteurization on various chemical characteristics of milk is investigated in detail, however, transition in antioxidant capacity of raw milk, chilled milk, pasteurized milk and long-term storage is not previously investigated. Some information is available regarding the antioxidant capacity of milk. Feed is the source of flavonoids, if the feed has a higher magnitude of flavonoids, milk will have higher concentration. In current investigation, flavonoids were estimated in terms of Rutin equivalent/ml. Flavonoids have antioxidant properties [36]. Total flavonoid content of the raw milk was 2.39 Rutin equivalent/ml. After 2 hrs of ambient storage, 18.41% flavonoids were lost. While the loss of total flavonoids in immediately and delayed chilled raw milk for 72 h was 7.53% and 9.62%, with no effect of pasteurization. After 3 days of storage of pasteurized immediately and delayed chilled raw milk, the loss of total flavonoids was 11.75% and 35.14% (Table 5). After 6 days of storage of pasteurized immediately and delayed chilled raw milk, the loss of total flavonoids was 18.41% and 46.86%. Khan et al. [37] studied the effect of pasteurization, boiling and storage period on flavonoid content of milk, they reported that heat treatments did not have a significant effect on total flavonoids content of milk, but the storage had a major effect on their concentration. Peroxide values and total flavonoid contents were strongly correlated, determination intervals showed a lower amount of total flavonoid content revealed the higher peroxide value. Total antioxidant capacity denotes the antioxidant status of milk and it is an important indication of the response of the milk to hostage the free radicals, this can be used good indication of antioxidant status of biochemical fluids [49]. In this investigation, total antioxidant capacity was determined to observe the antioxidant behaviour of raw, chilled, pasteurized milk at different stages of storage. Total antioxidant capacity and DPPH free radical scavenging activity of raw milk were 49.8% and 25.6%. Loss of 29.31% and 44.53 was recorded in total antioxidant capacity and DPPH free radical scavenging activity of milk, after 2 hrs of ambient storage. After 72 h of chilling of immediately and delayed chilled raw milk, loss of total antioxidant capacity was 8.43% and 37.55%. After 72 h of chilling of immediately and delayed chilled raw milk, loss of DPPH free radical scavenging activity was 8.2% and 53.1%. The effect of pasteurization treatment on total antioxidant capacity and DPPH free radical scavenging activity of milk was non-significant (*p* > 0.05). After 3 days of pasteurization, loss of total antioxidant capacity and DPPH free radical scavenging activity immediately chilled raw milk was 16.86% and 25.39%. After 3 days of pasteurization, loss of total antioxidant capacity and DPPH free radical scavenging activity delayed chilled raw milk was 40.56% and 54.29%. After 6 days of pasteurization, loss of total antioxidant capacity and DPPH free radical scavenging activity immediately chilled raw milk was 25.3% and 30.85%. After 6 days of pasteurization, loss of total antioxidant capacity and DPPH free radical scavenging activity delayed chilled raw milk was 72.1% and 89.57%. These results demonstrated that delayed chilling of raw milk is detrimental for the quality and shelf life of pasteurized milk.

**Table 5:** Effect of Immediate and Delayed Chilling of Raw Milk on Antioxidant Capacity of Pasteurized Milk

Stage of Sampling	TFC Rutin Equivalent mg/ml	TAC %	DPPH %
Raw Milk (Immediately Chilled After Milking)	2.39 ± 0.07^a^	49.8 ± 0.10^a^	25.6 ± 0.10^a^
Raw Milk Chilled After 2 Hrs of Storage (32 ± 1 °C)	1.95 ± 0.02^c^	35.2 ± 0.09^d^	14.2 ± 0.09^f^
Raw Milk Chilled for 72 Hrs (Immediately Chilled Milk)	2.21 ± 0.04^b^	45.6 ± 0.13^b^	23.5 ± 0.10^b^
Raw Milk Chilled for 72 Hrs (Delayed Chilled Milk)	1.72 ± 0.01^d^	31.1 ± 0.11^d^	11.9 ± 0.08^d^
0 Day After Pasteurization (Immediately Chilled Milk)	2.18 ± 0.04^b^	44.8 ± 0.12^b^	23.1 ± 0.11^b^
0 Day After Pasteurization Treatment (Delayed Chilled Milk)	1.69 ± 0.03^d^	29.6 ± 0.09^e^	11.7 ± 0.07^d^
After 3 Days of Storage (Immediately Chilled Milk)	2.11 ± 0.05^b^	41.4 ± 0.15^c^	19.1 ± 0.10^c^
After 3 Days of Storage (Delayed Chilled Milk)	1.55 ± 0.01^e^	21.9 ± 0.08^e^	6.95 ± 0.08^g^
After 6 Days of Storage (Immediately Chilled Milk)	1.93 ± 0.03^c^	37.2 ± 0.10^d^	17.7 ± 0.13^e^
After 6 Days of Storage (Delayed Chilled Milk)	1.27 ± 0.06^f^	13.9 ± 0.09^f^	2.67 ± 0.07^h^

In a column, if a mean is expressed by dissimilar letter, these are statistically significant (*p* < 0.05)

## Conclusions

The present study described that the lactose and pH of delayed chilled raw milk was less than immediately chilled milk while major changes were recorded in fatty acid profile of delayed chilled milk during the storage period of 6 days. Delayed chilled raw milk showed higher peroxide value and lower antioxidant capacity. In addition to several other factors, delayed chilling of raw milk may also be one of the most important reason for the shorter shelf life of pasteurized milk in developing countries. From this work, it was concluded that raw milk should be immediately cooled chilled for better shelf life. Therefore, it is recommended that milk collection system in the developing countries should be improved to avoid any delay in between milking and chilling of raw milk.
